# Cell death regulation by MAMs: from molecular mechanisms to therapeutic implications in cardiovascular diseases

**DOI:** 10.1038/s41419-022-04942-2

**Published:** 2022-05-27

**Authors:** Yiran E. Li, James R. Sowers, Claudio Hetz, Jun Ren

**Affiliations:** 1 Department of Cardiology, Zhongshan Hospital, Fudan University; Shanghai Institute of Cardiovascular Diseases, Shanghai, 200032 China; 2 Diabetes and Cardiovascular Center, University of Missouri School of Medicine, Columbia, MO 65212 USA; 3 Biomedical Neuroscience Institute (BNI), Faculty of Medicine, University of Chile, Santiago, Chile; 4 Program of Cellular and Molecular Biology, Institute of Biomedical Sciences, University of Chile, Santiago, Chile; 5 FONDAP Center for Geroscience, Brain Health and Metabolism, Santiago, Chile; 6 Buck Institute for Research in Aging, Novato, CA 94945 USA; 7 Department of Laboratory Medicine and Pathology, University of Washington, Seattle, WA 98195 USA

**Keywords:** Cardiovascular diseases, Cardiomyopathies

## Abstract

The endoplasmic reticulum (ER) and mitochondria are interconnected intracellular organelles with vital roles in the regulation of cell signaling and function. While the ER participates in a number of biological processes including lipid biosynthesis, Ca^2+^ storage and protein folding and processing, mitochondria are highly dynamic organelles governing ATP synthesis, free radical production, innate immunity and apoptosis. Interplay between the ER and mitochondria plays a crucial role in regulating energy metabolism and cell fate control under stress. The mitochondria-associated membranes (MAMs) denote physical contact sites between ER and mitochondria that mediate bidirectional communications between the two organelles. Although Ca^2+^ transport from ER to mitochondria is vital for mitochondrial homeostasis and energy metabolism, unrestrained Ca^2+^ transfer may result in mitochondrial Ca^2+^ overload, mitochondrial damage and cell death. Here we summarize the roles of MAMs in cell physiology and its impact in pathological conditions with a focus on cardiovascular disease. The possibility of manipulating ER-mitochondria contacts as potential therapeutic approaches is also discussed.

## Facts

- MAMs refer to membranous contact sites between ER and mitochondria, which mediate bidirectional communications between the two organelles.
- MAMs control cell homeostasis and cell death. The death signals governed by MAMs may be presented as excess Ca^2+^ transmission from ER to mitochondria, molecule translocation, protein-protein interaction or lipid metabolism.
- Changes in physiological events modulated by ER and mitochondria communication are common triggers for apoptosis, autophagy, necroptosis, pyroptosis and ferroptosis.
- Ca^2+^ ions either boost metabolism or evokes apoptosis. Ca^2+^ flux regulated by MAMs dictates the fate of cellular events.
- Dysregulation of MAMs functions is correlated with etiology of cardiovascular diseases.
- Targeting MAMs and associated components exhibits promises in therapeutic intervention of cardiovascular pathologies.

## Open Questions

- Identification of the functional significance of various components on MAMs to distinct the forms of cell death.
- Determination of second messages or metabolites other than Ca^2+^ ions transported through MAMs in cell death control?
- Development of small molecule drugs or gene therapy strategies directly targeting MAMs to improve cell physiology in disease conditions or prevent cell death.

## Introduction

The endoplasmic reticulum (ER) and mitochondria are tightly intertwined intracellular organelles in eukaryotic cells with a concerted role in the maintenance of cellular homeostasis. The ER is crucial to Ca^2+^ transport and storage, synthesis, transfer, modification and processing of lipids and proteins [1–3]. On the other hand, mitochondria serve as the “powerhouse” within the cell to offer a stable energy supply for cellular function and cell survival. Reminiscent of other membrane organelles including ER, mitochondria are highly dynamic structures with disparate composition, morphology and location in various cell types [4–7]. Ample evidence has depicted the existence of structural and functional contacts between mitochondria and other organelles, including the ER-mitochondria coupling [1, 8–10]. Mutually coupled membrane components between mitochondria and ER (referred to as mitochondria-associated membranes, MAMs) have been observed using fluorescence and electron microscopy, and were characterized using subcellular fractionation and biochemistry [11, 12]. As the name suggests, MAMs refer to the complex of outer mitochondrial membrane (OMM) and ER segregated by a distance of 10–40 nm, comprising tether proteins, Ca^2+^ transfer proteins and lipids. It is not surprising that such membranous association facilitates the interaction between ER and mitochondria. The ER may respond to various physiological or pathophysiological stimuli to transfer ions, lipids and protein signals into mitochondria, resulting in a fine-tuning of mitochondrial physiology. On the other side of the coin, the mitochondria may also deliver ions, lipid or protein signals to the ER [13–15].

As mentioned earlier, MAMs control not only cell homeostasis but also cell death events. The cell death signals governed by MAMs may be presented in various forms, including Ca^2+^ transmission from ER to mitochondria, molecule translocation, protein-protein interaction and lipid metabolism. Mitochondrial permeability transition pores (mPTPs), a high-conductance channel composed of adenine nucleotide translocase (ANT) in the inner mitochondrial membrane (IMM), the voltage-dependent anion channel (VDAC) resided in the OMM and cyclophilin D in the matrix, is the main driver of cell death [16]. Since MAM serves as a unique platform for numerous physiological activities, even subtle or mild disruptions of their structure might evoke a series of pathological sequelae including Alzheimer disease, Parkinson disease, cancer, cardiovascular diseases and metabolic disease (e.g., obesity and diabetes mellitus) [17–19]. In particular, interruption or deficiency in ER-mitochondria communication seems crucial in the pathogenesis of cardiovascular diseases such as ischemia-reperfusion (I/R) injury, heart failure, pulmonary hypertension, and atherosclerosis, which underscore an essential role and therapeutic promises for MAMs in the management of these pathological conditions [9, 20]. Therefore, in this review, we will focus on the molecular aspects of MAMs, components and regulatory mechanisms in cell death. We will also explore how abnormalities in ER-mitochondria contacts may be linked with the highly prevalent cardiovascular diseases.

## MAMs components

MAMs are deemed an independent specialized cell compartment where multiple biological events take place, including Ca^2+^ signaling, lipid metabolism, mitochondrial dynamic alteration and apoptosis [21]. The composition of MAMs is highly dynamic and includes over 1000 proteins that participate in these cellular processes [22]. Without direct fusion of their membranes, the ER and mitochondria maintain a stable but dynamic communication courtesy of proteins that form tethers between the two organelles. Components of MAMs can be classified based on their ability to promote or prevent tethering or their functional impact on organelle homeostasis [2]. These effects are non-exclusive because certain MAM components can modulate cell functional properties in the absence of any apparent ultrastructural alteration [2]. However, conditions that disrupt MAM morphology would usually impact several mitochondrial and ER functions.

MAMs perform biological functions through a number of protein complexes with not only tethering property but also unique functionality [23]. Recognition and characterization of proteins involved in MAMs are an expanding field of research and drug development. Proteins residing within MAMs can be categorized into three classes namely: (1) proteins specifically targeted to MAMs; (2) proteins also present in other organelles; and (3) proteins translocated to MAMs temporarily under diverse cell stimuli [22]. In this section, we will hereby mainly focus on the MAM protein complexes which would enable physical and functional interplay between ER and mitochondria, and those proteins/molecules translocated onto MAMs during cell death process.

### IP_3_R-GRP75-VDAC complex

**Fig. 1:**
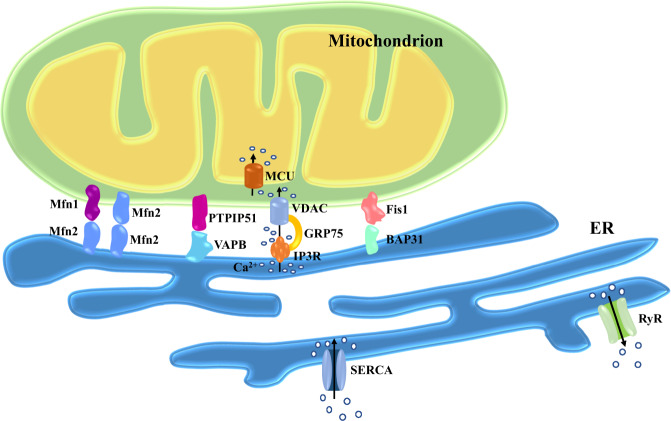
Main tethering proteins involved in the maintenance of MAMs. Mitochondria are connected to ER by protein complexes. ER-resident Mfn2 forms hetero- or homodimers with Mfn1/2 in OMM. The ER protein VAPB interacts with the mitochondrial protein PTPIP51. The ER-localized IP3R partners with the mitochondrial protein VDAC through GRP75. BAP31 resided in the ER is anchored to the OMM-localized Fis1. SERCA is the main pump responsible for Ca^2+^ uptake in the ER. RyR releases Ca^2+^ from ER into the cytoplasm. The IMM-resident MCU is the predominant pathway transferring Ca^2+^ into mitochondrial matrix. Abbreviations: BAP31, B-cell receptor-associated protein 31; ER, endoplasmic reticulum; Fis1, fission protein 1 homolog; GRP75, glucose-regulated protein 75; IMM, inner mitochondrial membrane; IP3R, inositol 1,4,5-triphosphate receptors; MAMs, mitochondria-associated membranes; MCU, mitochondrial Ca^2+^ uniporter; Mfn1/2, mitofusin-1 and -2; OMM, outer mitochondrial membrane; PTPIP51, protein tyrosine phosphatase-interacting protein-51; RyR, ryanodine receptor; SERCA, sarco(endo)plasmic reticulum calcium-ATPases; VAPB, vesicle-associated membrane protein-associated protein-B; VDAC, voltage-dependent anion channels.

Inositol 1,4,5-triphosphate receptors (IP_3_Rs) are vital Ca^2+^ outflow channels located on the ER surface mediating Ca^2+^ transfer from the ER lumen into cytoplasm. Elevated IP_3_Rs levels or activity are common in pathological conditions including hypertension, cardiac hypertrophy, ischemic dilated cardiomyopathy and heart failure [24, 25]. VDACs refer to ion channels localized on the OMM to regulate the transfer of metabolites and ions across mitochondrial membranes. VDACs participate in multiple cellular function including metabolism, apoptosis and Ca^2+^ flux. It was shown that VDACs promotes the bridging or connectivity between ER and mitochondria to facilitate Ca^2+^ flux into mitochondria. Silencing or inhibition of VDAC1 exhibits a loosened ER-mitochondria contact [26]. Glucose-regulated protein 75 (GRP75) bridges IP_3_Rs with VDACs to sustain MAMs architecture, with higher levels of GRP75 favoring a higher degree of IP_3_R-VDAC interaction. This is in line with the fact that silencing GRP75 disrupts the connection between IP_3_Rs and VDAC (Fig. 1) [27, 28].

### Fis1–BAP31 complex

The protein complex ARCosome consisting of the mitochondrial fission protein 1 homolog (Fis1) and B-cell receptor-associated protein 31 (BAP31) not only carries tethering capacity, but also transfers apoptotic signals between the two organelles (Fig. 1) [29]. ARCosome participates in the regulation of cellular stress, which gives rise to recruitment and stimulation of procaspase 8. An interaction with caspase-8 alters ARCosome configuration by cleaving BAP31 to form proapoptotic p20BAP31, which transfers Ca^2+^ from ER to mitochondria, triggering apoptotic signal through IP_3_R complex in MAMs [30, 31]. In addition, BAP31 is vital for mitochondrial O_2_ consumption, autophagy and mitochondrial homeostasis through facilitated formation of the mitochondrial complex I. BAP31 is involved in sepsis-mediated myocardial defect, the effect of which was ameliorated by melatonin [32]. Fis1 plays an important role in mitochondrial dynamics and regulates dynamin-related protein 1 (Drp1) oligomerization and translocation to mitochondria for mitochondrial fission. Interaction of Fis1 with mitofusin-1/-2 (Mfn1, Mfn2) has been shown to suppress their GTPase activity and inhibit the mitochondrial fusion process [33, 34].

### Mfn1–Mfn2 complex

In addition to its localization in OMM and perceived role in mitochondrial fusion, the GTPase homologous protein Mfn2 also resides on the ER surface. Mfn2 is imperative for the maintenance of MAMs structural by forming hetero- or homodimers with Mfn1/2 in OMM (Fig. 1). This connection controls not only distance between organelles, but also coordination of ER-mitochondria dynamics [35, 36]. Although this commonly accepted model has been well-documented, several studies have yielded contradictory results [9]. For example, ablation of Mfn2 increases the proximity between the two organelles and promotes Ca^2+^ transmission from the ER to mitochondria, suggesting a role of Mfn2 as a tethered antagonist preventing excessive connect [37, 38]. Furthermore, Mfn2 was suggested to possess a role in ER stress responsiveness. Downregulation of Mfn2 was noted in cardiac hypertrophic models including spontaneous hypertension, myocardial infarction (MI) and transverse aortic banding, all of which led to unfavorable myocardial remodeling. However, upregulation of Mfn2 mitigates angiotensin II-inducing cardiac hypertrophy [39]. Moreover, Mfn2 is essential to cardiac differentiation of embryonic stem cells [40].

### VAPB–PTPIP51 complex

The OMM protein, tyrosine phosphatase-interacting protein-51 (PTPIP51), which presides over the unfolded protein response and vesicle trafficking, is recognized as a binding partner for vesicle-associated membrane protein-associated protein-B (VAPB) - an ER-resident protein regulating cell development and tumorigenesis (Fig. 1) [41, 42]. Disrupted connection between PTPIP51 and VAPB leads to dissociation of MAMs, disturbance of mitochondrial Ca^2+^ import and ATP production [43]. Indeed, overexpressed PTPIP51 contributes to a collapse in mitochondrial membrane potential and enhanced cytochrome C release, ultimately resulting in apoptosis [44]. Moreover, VAPB ablation gives rise to the accumulation of mitochondria and compromised Ca^2+^ processing capacity. Downregulation of either PTPIP51 or VAPB stimulates autophagy through reduction in mitochondrial Ca^2+^, while excessive autophagy causes further myocardial damage under I/R challenge [45, 46].

## MAMs and cell death

Communication between ER and mitochondria modulates multiple physiological events, including Ca^2+^ homeostasis, lipid biosynthesis and trafficking as well as protein-protein crosstalk control. Changes in these processes are deemed common triggers of apoptosis, autophagy, necroptosis, pyroptosis and ferroptosis, indicating a crucial role for MAMs in the regulation of cell death [21]. Here we will discuss these functions in more details and summarize the vital roles of MAMs and associated proteins in Table 1.

**Table 1:** Components of MAMs participated in cardiovascular diseases.

Proteins	Relevant functions in MAMs	Functions in CVD
IP3Rs	Ca^2+^ outflow channels located on the ER surface mediating Ca^2+^ transfer in MAMs.	IP3Rs downregulation alleviates mitochondrial Ca^2+^ overload, myocardial cell death and infarct area in I/R hearts [24, 25, 102].
GRP75	Bridges IP3Rs to VDACs to sustain MAMs structure.	Mitochondrial Ca^2+^ overload and H/R injury in myocardial cells [27, 28, 100].
VDACs	Ion channels on OMM regulate movement of metabolites and ions across mitochondria.	Level of VDACs elevates in myocardial infarction [26].
Fis1	Regulates ER-mitochondria tethering, apoptosis, and mitochondrial dynamics.	Disruption of Fis1-mediated signaling results in heart diseases [29, 33, 34].
Mfn2	Modulator of MAMs structure by forming hetero- or homodimers with Mfn1/2. Responds to ER stress.	Mfn2 decreases in cardiac hypertrophy. High Mfn2 inhibits cardiac hypertrophy and phenotypic switching in PASMCs [35, 36, 39, 134].
Mfn1	Tethering mitochondria to ER through connection with ER-located Mfn2	Mfn1 knockout is effective in relieving cardiac hypertrophy and I/R injury [35, 36, 107].
VAPB	Physically/functionally interacts ER to mitochondria through OMM-resident PTPIP51. Modulates mitochondrial Ca^2+^ level.	Downregulation of VAPB elicits myocardial damage during cardiac I/R [41, 42, 45, 46].
PTPIP51	Physically/functionally interacts mitochondria to ER via connecting to ER-resident VAPB. Modulates mitochondrial Ca^2+^ level.	Overtly elevated PTPIP51 in cardiomyocytes following I/R [41, 42, 44].
Sig-1R	Reduces IP3Rs degradation under ER stress and boosts Ca^2+^ transmission to mitochondria.	Sig-1R inhibition promotes autophagy in cardiomyocytes under oxidative stress whereas its stimulation represses hypertrophy and myocardial cell injury [65, 115, 116].
FUNDC1	Facilitates mitochondrial fission and mitophagy. Regulates mitochondrial Ca^2+^ level.	FUNDC1 decreases in HF. Heart-specific FUNDC1 ablation exhibits interstitial fibrosis, compromised cardiac function and elevated apoptosis [67, 85, 112–114].
mPTP	Promotes Ca^2+^-dependent apoptosis.	Mediator of cardiomyocyte death during reperfusion damage [20, 55–57].
PACS2	Favors ER-mitochondria coupling and controls Bid-mediated apoptosis.	PACS2 silencing promotes VSMCs apoptosis and plaque rupture [70, 71, 137].
Drp1	Modulates mitochondrial dynamics, apoptosis and mitophagy.	Drp1 overexpression evokes cardiac hypertrophy and phenotypic change in PASMCs [33, 34, 74, 75, 85, 123, 134].
CypD	Regulates Ca^2+^ transfer from ER to mitochondria via IP3R.	Inhibition of CypD-IP3R-GRP75-VDAC complex protects mitochondrial Ca^2+^ overload and H/R myocardial cells [97, 100].

*Bid* BH3-interacting domain death agonist, *CVD* cardiovascular diseases, *CypD* Cyclophilin D, *Drp1* dynamin-related protein 1, *ER* endoplasmic reticulum, *Fis1* fission protein 1 homolog, *FUNDC1* FUN14 domain containing 1, *GRP75* glucose-regulated protein 75, *HF* heart failure, *H/R* hypoxia/re-oxygenation, *IP3R* inositol 1,4,5-triphosphate receptors, *I/R* ischemia-reperfusion, *MAMs* mitochondria-associated membranes, *Mfn1/2* mitofusin-1/−2, *mPTP* mitochondrial permeability transition pores, *OMM* outer mitochondrial membrane, *PACS2* phosphofurin acidic cluster sorting protein 2, *PASMCs* pulmonary artery smooth muscle cells, *PTPIP51* protein tyrosine phosphatase-interacting protein-51, *Sig-1R* sigma-1 receptor, *VAPB* vesicle-associated membrane protein-associated protein-B, *VDACs* voltage-dependent anion channels, VSMCs vascular smooth muscle cells.

### Ca^2+^ signaling in apoptosis

#### Physiological role of Ca^2+^

Ca^2+^ is crucial for communication between ER and mitochondria, and its level is tightly maintained by the cytosolic Ca^2+^ reservoir – sarcoplasmic reticulum (SR) or ER through Ca^2+^ reuptake pump sarco(endo)plasmic reticulum Ca^2+^-ATPases (SERCAs). SERCAs manifest in several subtypes, of which the ubiquitous SERCA2b possesses the highest affinity for cytoplasmic Ca^2+^ [47]. SR/ER releases bursts of Ca^2+^ ions in response to electrical excitation or pharmaceutical stimulation of the ryanodine receptor (RyR). Ca^2+^ can move freely through the OMM with the assistance of VDACs, whereas the IMM is not permeable and Ca^2+^ can only influx through the mitochondrial Ca^2+^ uniporter (MCU) with a relatively weak affinity to cytosolic Ca^2+^ [48]. However, local Ca^2+^ levels around SR/ER are considerably higher than global cytosolic Ca^2+^. Given the vicinity of mitochondria to SR/ER Ca^2+^ stores, MCU would exhibit a much higher affinity to SR/ER Ca^2+^ [49]. Moreover, proteins such as the mitochondrial calcium uptake (MiCU) family, SLC25A23 and MCUR1 all display regulatory capacity for MCU to impact the mitochondrial Ca^2+^ uptake efficiency [50]. Through suppression of disturbed SR/ER-mitochondria connection and stress within ER/SR, chemical chaperones including tauroursodeoxycholic acid and 4-phenylbutyrate (PBA) were found to alleviate established pulmonary arterial hypertension (PAH) in rodents [51].

**Fig. 2:**
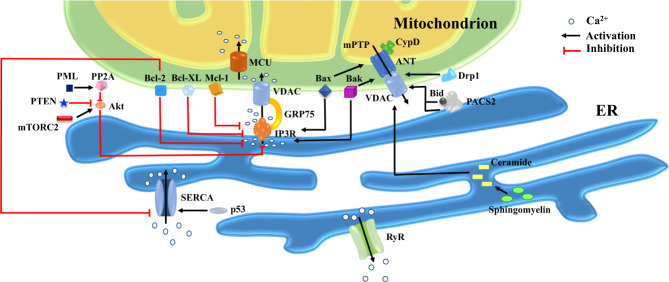
Representation of principle pro- and anti-apoptotic molecules at MAMs. Death signals governed by MAMs may be present in various forms: 1. ER perfuses Ca^2+^ into mitochondria and massive Ca^2+^ in mitochondria serves as an initial instigating signal for cell death. Multiple factors alter Ca^2+^ levels in mitochondria via directly or indirectly action on the IP3R-GRP75-VDACs complex, for example, anti-apoptotic members of Bcl-2 family (e.g., Bcl-2, Bcl-XL and Mcl-1) restrain Ca^2+^ release by directly modulating IP_3_R activity, while molecules such as mTORC2, PTEN and PML regulate IP_3_R-mediated Ca^2+^ efflux indirectly by acting on Akt. Bcl-2 and p53 modulate Ca^2+^ enrichment in the ER via altering SERCA activity; 2. PACS2 translocates from ER to mitochondria along with the translocation and activation of Bid, leading to increased permeability of OMM and release of pro-apoptotic factors. Other mitochondrially-targeted players including Bax/Bak and Drp1 can also trigger mPTP opening and subsequently initiation of apoptosis; 3. Ceramide synthesis and collection contribute to pores formation across the OMM, inducing cytosolic release of pro-apoptotic factors and stress signal delivery from ER to mitochondria. The ER-mitochondria-ER amplification loop of pro-apoptotic signals helps to coordinate death events between these two organelles. Abbreviations: Akt, serine-threonine protein kinase; ANT, adenine nucleotide translocase; Bax, Bcl-2-associated X protein; Bak, Bcl-2 antagonist killer; Bcl-2, B-cell lymphoma 2; Bcl-XL, B-cell lymphoma-extra large; Bid, BH3-interacting domain death agonist; CypD, Cyclophilin D; Drp1, dynamin-related protein 1; ER, endoplasmic reticulum; GRP75, glucose-regulated protein 75; IP3R, inositol 1,4,5-triphosphate receptors; Mcl-1, myeloid cell leukemia factor-1; MCU, mitochondrial Ca^2+^ uniporter; mPTP, mitochondrial permeability transition pores; mTORC2, mammalian target of rapamycin complex 2; PACS2, phosphofurin acidic cluster sorting protein 2; PML, promyelocytic leukemia protein; PP2A, protein phosphatase 2A; PTEN, phosphatase and tensin homolog; RyR, ryanodine receptor; SERCA, sarco(endo)plasmic reticulum calcium-ATPases; VDAC, voltage-dependent anion channels.

As shown in Fig. 2, effective Ca^2+^ transfer from the ER to the mitochondria is mainly regulated by the IP_3_Rs-GRP75-VDACs complexes. When ER-resident Ca^2+^ release channels (IP_3_Rs) open, a high Ca^2+^ microdomain can be formed within the ER vicinity. This prompts Ca^2+^ uptake by VDACs localized on the OMM [52]. The third component of the complex, GRP75, links two channels through their cytosolic portions to build the IP_3_Rs-GRP75-VDACs channel complex [27]. In this manner, Ca^2+^ is directly transmitted from the ER to cytoplasm and cross OMM, subsequently into mitochondrial matrix through MCU [2, 49].

Existence of Ca^2+^ in mitochondrial matrix has various functions. Firstly, it can improve the efficiency of tricarboxylic acid (TCA) cycle and the electron transport chain (ETC), thus facilitating ATP production [53]. Ca^2+^ ions that enter mitochondria boost energy metabolism by favoring the enzymatic activity of key rate limiting dehydrogenases of the TCA cycle, including isocitrate dehydrogenase (IDH), pyruvate dehydrogenase (PDH), oxoglutarate dehydrogenase (OGDH or α-KGDH), and glycerol-3-phosphate dehydrogenase (GPD1/G3PDH) [48]. In addition, Ca^2+^ may control metabolism by modulating glucose transporter activity [54]. However, Ca^2+^ serves as a double-edged sword. Ca^2+^ ions may boost cell metabolism or evoke cell death. Thus Ca^2+^ flux as the main Ca^2+^ conveyance dictates in occurrence of these cellular events, thus supporting a role for MAMs in cell fate control [48]. High level and long-lasting Ca^2+^ overload result in cell death. Ca^2+^ interacts with cyclophilin D and ANT to form mPTP. Excess mitochondrial Ca^2+^ uptake evokes mPTP opening, mitochondrial swelling and rupture of the OMM. OMM rupture is responsible for release of pro-apoptotic factors such as cytochrome C (Cyt c) and apoptosis inducing factor (AIF) [55–57]. Moreover, Ca^2+^ overload facilitates disassembly of respiratory chain complex II through binding to cardiolipin in the IMM, thus prompting release of various subunits. Such changes trigger substantial reactive oxygen species (ROS) production and eventually apoptosis [58]. Ample evidence has depicted the benefit of antioxidant such as N-acetylcysteine in the combat of mitochondrial damage and muscle dysfunction through amelioration of ROS and apoptosis [59].

#### Regulatory effect of MAMs on Ca^2+^ transfer

Given that mitochondrial Ca^2+^ is necessary to induce apoptosis, it is not surprising that factors affecting ER-to-mitochondria Ca^2+^ transfer positively or negatively influence this process of programmed cell death. Multiple factors alter Ca^2+^ levels in mitochondria via direct or indirect action on the IP_3_R-GRP75-VDACs complex (Fig. 2). For example, the serine-threonine protein kinase (Akt) in MAMs phosphorylates IP_3_R, thereby decreasing Ca^2+^ release from the ER and reducing cellular sensitivity to Ca^2+^-dependent apoptosis [60]. The mammalian target of rapamycin complex 2 (mTORC2) increase Akt activity through phosphorylation and consequently controls IP_3_R function, whereas phosphatase and tensin homolog (PTEN) can inhibit Akt and promote Ca^2+^ mobilization in MAMs, ultimately sensitizing cells to apoptotic stimuli [61]. Furthermore, promyelocytic leukemia protein (PML) may indirectly regulate IP_3_R phosphorylation via counteracting Akt through recruitment of protein phosphatase 2 A (PP2A) at the MAMs, which then induces cell death by mediating Ca^2+^ transfer into mitochondria [62]. In addition, B-cell lymphoma 2 (Bcl-2) and B-cell lymphoma-extra large (Bcl-X_L_), two Bcl-2 family members on MAMs, connect with central modulatory domain of IP_3_Rs to suppress Ca^2+^ release or indirectly inhibit IP_3_Rs through regulating its phosphorylation. Besides, both Bcl-2 and Bcl-X_L_ can conjugate with VDAC1 to suppress mitochondrial Ca^2+^ intake [63]. Moreover, myeloid cell leukemia factor-1 (Mcl-1), another anti-apoptotic protein of Bcl-2 family, not only restrains IP_3_R-mediated Ca^2+^ release but also binds with VDACs to modulate mitochondrial Ca^2+^ uptake. To the contrary, Bcl-2-associated X protein (Bax) and Bcl-2 antagonist killer (Bak), pro-apoptotic proteins from Bcl-2 family, regulate ER Ca^2+^ levels by binding to and displacing Bcl-2 from IP_3_R1, where the anti-apoptotic protein regulates phosphorylation status of IP_3_R1 and Ca^2+^ efflux from ER [64]. IP_3_Rs degradation may be suppressed by Sigma-1 receptor (Sig-1R), a vital component of MAMs. Sig-1R can be dislodged from binding Ig protein (Bip) and binds with IP_3_Rs under conditions of mitochondrial stress, thereby inhibiting degradation of IP_3_Rs to boost Ca^2+^ transmission into mitochondria [65, 66]. Moreover, FUN14 domain containing 1 (FUNDC1) resided in MAMs can maintain cardiac function under stress. By binding to F-box/LRR-repeat protein 2 (FBXL2), it promotes the degradation of IP_3_Rs and alleviates mitochondrial Ca^2+^ overload, thus dampening the sensitivity to Ca^2+^-dependent apoptosis [67].

SERCAs, the main pump responsible for Ca^2+^ uptake in the ER, cluster in MAMs where multiple proteins modulate its function orchestrating apoptosis (Fig. 2). With re-localization in MAMs, p53 alters SERCAs redox state and stimulates its function, consequently increasing ER Ca^2+^ levels and Ca^2+^ transfer to mitochondria to evoke Ca^2+^ overload. Moreover, Bcl-2 can directly interact with SERCAs to initiate a conformational change and downregulate its activity, thus suppressing Ca^2+^ enrichment in the ER [16]. Moreover, ER-to-mitochondria Ca^2+^ transfer can be modulated by the distance between ER and OMM at the MAMs. If the gap is around 15 nm between ER and mitochondria, it results in increased Ca^2+^ transfer efficiency. If the gap is limited to 5 nm, it causes reduced efficiency of Ca^2+^ transfer [68]. Except for the aforementioned GTPase Mfn-2, the spacer protein fetal and adult testis expressed 1 (FATE1) can decrease the level of contacts between ER and mitochondria to reduce Ca^2+^ flux with an impaired sensitivity to Ca^2+^-dependent apoptosis [69].

### Other players governing apoptosis between ER and mitochondria

The phosphofurin acidic cluster sorting protein 2 (PACS2) is a sorting protein located on ER surface engaged in the modulation of lipid metabolism, ER homeostasis and ER-mitochondria tethering. Besides, PACS2 participates in apoptotic signaling in MAMs. High levels of ER stress evoke dephosphorylation of the full-length BH3-interacting domain death agonist (Bid) and its connection with PACS2, fostering Bid-bound PACS2 to translocate from ER to mitochondria where Bid is cleaved by caspase-8 on mitochondria producing truncated form of Bid (tBid) to bind with Bax/Bak, leading to increased permeability of OMM, release of cytochrome c and initiation of apoptosis (Fig. 2) [70, 71]. In addition, Nip3-like protein X (Nix), a BH3-only-like protein of Bcl-2 family, induces cell death depending on whether it is mitochondria- or ER-resided. Mitochondrially-targeted Nix causes Bax/Bak-dependent OMM permeabilization, cytochrome c release, caspase activation and apoptosis. Contrarily, Nix targeted to the ER triggers mPTP opening, leading to loss of ATP, mitochondrial swelling, OMM rupture and cytochrome c release. These changes feature necroptosis, a form of regulated necrotic cell death [72, 73].

Furthermore, mitochondrial fission protein Mfn2 may participate in apoptosis by linking to BAP31, a chaperone localized on ER surface to produce p20 BAP31 [29]. As a pro-apoptotic protein, p20 BAP31 converts procaspase-8 into activated form to tun on apoptosis. Activation of p20 BAP31 facilitates Ca^2+^ transfer from the ER into mitochondria, suggesting a recycling of apoptotic signals back to mitochondria, forming an amplified loop of apoptosis to coordinate activities between the two organelles [30]. Interestingly, mitochondrial fission is elevated during apoptosis due to abundant recruitment of Drp1 from the cytoplasm onto OMM. Prominently, transfection of dominant negative Drp1 mutant mitigates apoptosis, unveiling the link between mitochondrial fission and apoptosis. In addition, Drp1 promotes Bax oligomerization, thereby favoring apoptosis [74, 75]. Moreover, Drp1 also plays a role in cardiomyocyte pyroptosis, a proinflammatory form of regulated cell death in the defense of exogenous pathogens including bacteria, virus and fungi. Mechanistically, the anthracycline antibiotics doxorubicin promotes nicotinamide adenine dinucleotide phosphate oxidases (NOX)1 and NOX4 expression and induces mitochondrial fission by Drp1 activation, resulting in NACHT, LRR, and PYD domain containing protein 3 (NLRP3) inflammasome-mediated pyroptosis in cardiomyocytes through caspase-1-dependent pattern. These findings denote the promises for NLRP3 inflammasome and pyroptosis as therapeutic targets in the management of anthracycline cardiotoxicity [76, 77] although whether MAMs participate in the regulation of pyroptosis remains unknown.

### MAMs and autophagy

MAMs are closely associated with autophagy, or generation of double-membrane vesicles named autophagosomes. Although the derivation of autophagosomal membrane is still elusive, MAMs are suggested to be the main sites of autophagosome formation [78]. The recruitment of the pre-autophagosome marker autophagy-related 14-like (ATG14L) resided in MAMs can trigger autophagosome biogenesis, Drp1 and ATG14L further promote enrichment of different autophagy-related proteins in MAMs [79, 80]. The integrity of MAMs is essential for the formation of autophagosomes. Mfn2 depletion was shown to alter the ER-mitochondria contact sites accompanied with impaired autophagosome generation. Moreover, as the major regulator of autophagy, mTORC2 resides in MAMs [81]. Gomez-Suaga and colleagues reported that MAMs tethering constituted by VAPB-PTPIP51 complex regulates autophagy, courtesy of its capacity to modulate MAMs Ca^2+^ transfer. Overexpression of either one of these proteins tightens ER-mitochondria communication, resulting in diminished autophagosome formation by Ca^2+^ flux promotion, whereas an opposite effect on autophagosome formation is identified when any of the two proteins is silenced [46].

The most common form of selective autophagy is mitophagy, which particularly aims at impaired or excess mitochondria to degrade. Dysfunctional mitochondria are initially isolated from mitochondrial network through fission, and are subsequently fused with lysosomes for degradation by autophagic mechanism [82]. PTEN-inducible putative kinase 1-Parkinson juvenile disease protein 2 (PINK1-Parkin) mediated mitophagy is most studied. PINK1 located in OMM of impaired mitochondria facilitates Parkin translocating from the cytoplasm to OMM, which ubiquitinates OMM proteins including Mfn2 and VDAC. These ubiquitinated proteins will be identified by the autophagosomal membrane involving microtubule associated protein 1 light chain 3 (LC3) and sequestosome 1 (p62), therefore facilitating mitochondrial degradation [83, 84]. Enhancing the link with mitophagy mechanism, hypoxia-induced FUNDC1 resided in OMM has been reported to serve as mitophagy receptor, recruiting autophagosomes and resulting in mitochondrial degradation in response to hypoxia. Moreover, FUNDC1 also recruits Drp1 at MAMs, propelling mitochondrial fission and mitophagy under hypoxia [85]. All these observations strengthen the association between ER-mitochondria interplay and autophagy.

### The relationship between lipid metabolism and cell death

MAMs play a crucial role in lipid synthesis and host multiple key enzymes in phospholipid biosynthesis. Certain lipid metabolites can influence cell fate, among which ceramide is the most typical prototype. In normal conditions, ceramide is synthesized using ceramide synthase pathway. Nevertheless, ceramide is rapidly synthesized from sphingomyelin by sphingolipase under stress circumstances [86, 87]. Aggregation of ceramide directly or indirectly modulates molecules involved in apoptosis including Ras, protein kinase C and protein phosphatase 1 A/2 A. Buildup of ceramide also contributes to pore formation across the OMM inducing cytosolic release of pro-apoptotic factors, such as cytochrome c, and delivers stress signal from the ER to mitochondria (Fig. 2) [88]. In addition, ceramide is a second messenger with potent pleiotropic property in necroptosis, such as lipid peroxidation, nitric oxide synthase activation and mitochondrial ROS production [89]. Given the role of mitochondrial ceramide in apoptosis and necroptosis, MAMs may act as a pivotal checkpoint to avoid mitochondrial ceramide uptake, thereby governing cellular lifespan shifts.

Moreover, depending on the type of phospholipids being oxidized, death signals produced at MAMs might induce either apoptosis or ferroptosis, a form of ROS-dependent iron-regulated cell death featured by lipid peroxidation [90]. Although direct relationship between MAMs and ferroptosis has not been elucidated, recent observations from quantitative redox lipidomics indicated oxidation of polyunsaturated lipids (primary ferroptosis executors) within ER, such as oxidation of phosphatidylethanolamine by adrenoyl (AdA) or arachidonoyl (AA) fatty acyls [91]. Especially, acyl-CoA synthetase long-chain family member 4 (ACSL4), which catalyzes AA biosynthesis to facilitate esterification in phospholipids and ferroptosis, was reported to accumulate in MAMs [92]. Hence, these findings support a possible link between MAMs and ferroptosis.

In addition, MAMs lipid raft constitution is perceived as a vital player in autophagic process. These microdomains promote initial organelle crosstalk, resulting in autophagosomes formation [93]. Bosc and team reported a bidirectional trafficking model between mitochondria lipid metabolism and autophagy, where mitochondria modulate free fatty acid supply through formation of autophagosome in MAMs. Such connection enables the structural and functional communication with free fatty acid supply from lipid droplet to fuel TCA cycle in neighboring mitochondria [94].

## Implication of MAMs-regulated cell death in cardiovascular disease and therapeutics

### Ischemia–reperfusion (I/R)

I/R injury evokes ER stress and mitochondrial damage in cardiomyocytes, contributing to high prevalence of coronary artery diseases [7, 95]. In cardiac I/R injury, degradation of long-lived or impaired mitochondria through mitophagy is vital to sustain myocardial fitness. I/R challenge dampens mitophagy and facilitates apoptosis in ischemic myocardium. This is supported by relief of I/R-induced apoptosis with proper elevation in mitophagy [7, 96].

MAMs possess a key role in managing mPTP opening, a main mediator of cardiomyocyte death during reperfusion damage [20]. Cyclophilin D resided in the mitochondrial matrix plays a vital role in regulating mPTP opening and necrosis. Overexpression of cyclophilin D induced mPTP opening without any inciting death stimulus. In contrast, absence of cyclophilin D may render mitochondria resistant to Ca^2+^-induced mPTP opening and protect cells against necrotic stimuli [50, 73, 97]. Indeed, deletion of cyclophilin D significantly reduced infarct size during I/R [97–99]. The CypD-IP_3_R-GRP75-VDAC complex mainly modulates cation exchange in MAMs. Connection of this complex reduces with suppression of its compositions, leading to relief of mitochondrial Ca^2+^ overload and protection against hypoxia/reoxygenation (H/R) damage in cardiomyocytes [100]. Moreover, glycogen synthase kinase 3β (GSK-3β) resided in MAMs connects with CypD-IP_3_R-GRP75-VDAC complex, and this interaction strengthens along with cell death following H/R injury [101]. Inhibition of GSK-3β compromised IP_3_R function, abolished mitochondrial Ca^2+^ overload and alleviated cell death as well as infarct area in reperfused hearts [102]. In accordance with these observations, utilization of pharmacological reagents and small interfering RNA (siRNA) technique targeting VDAC1 may prevent GSK-3β from being translocated to mitochondria and ultimately mPTP opening under cellular stress.

PTPIP51, a mitochondria-resident protein, facilitates mitochondrial Ca^2+^ influx and apoptosis via widening the proximity between SR and mitochondria. Qiao and colleagues noted overtly elevated PTPIP51 in mouse hearts following I/R challenge, while knockout of PTPIP51 markedly ameliorated I/R-induced cardiomyocyte dysfunction and infarct size [45]. Nevertheless, the precise role for PTPIP51 remains poorly defined in I/R injury other than its perceived ER-mitochondria tethering action. Patricia and coworkers elucidated that downregulation of either PTPIP51 or VAPB elicited autophagy by lowering mitochondrial Ca^2+^ to protect against I/R injury although excess autophagy in reperfusion triggered further myocardial injury [103]. Hence, mechanism underlying the protective role of PTPIP51 suppression in myocardial ischemia deserves in depth scrutiny. Given that SERCA is negatively correlated with cytoplasmic Ca^2+^, mitochondrial fission and mitochondrial ROS in cardiomyocytes, maneuvers which regulate SERCA function may be closely associated with myocardial susceptibility to I/R challenge. For instance, transmembrane protein related to thioredoxin 1 (TMX-1) and FUNDC1 were shown to attenuate the sensitivity of the heart to reperfusion damage through modulation of SERCA function [85, 104].

**Fig. 3:**
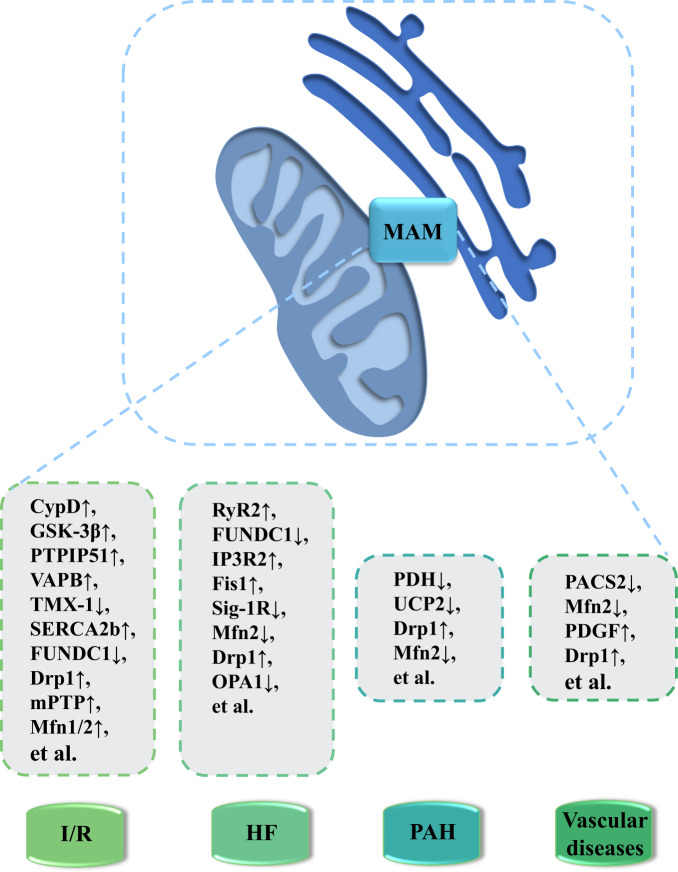
MAM-enriched proteins as potential new therapeutic targets for the treatment of CVD pathologies. Disruption or deficiency in ER-mitochondria communication is crucial in the pathogenesis of various CVDs such as I/R, HF, PAH and vascular diseases. For example, expression level of CypD, PTPIP51 and mPTP is elevated in I/R, whereas downregulation of FUNDC1, Sig-1R and OPA1 is noted in HF. Drp1 level is increased while Mfn2 level is decreased in PAH and vascular diseases. Notably, the key regulatory proteins of these processes serve as promising therapeutic targets in the management of these pathological conditions. Abbreviations: CVD, cardiovascular diseases; CypD, Cyclophilin D; Drp1, dynamin-related protein 1; Fis1: fission protein 1 homolog; FUNDC1, FUN14 domain containing 1; GSK-3β, glycogen synthase kinase 3 beta; HF, heart failure; IP3R2, inositol 1,4,5-triphosphate receptors 2; I/R, ischemia-reperfusion; MAM, mitochondria-associated membrane; Mfn1/2, mitofusin-1 and -2; mPTP, mitochondrial permeability transition pores; OPA1, optic atrophy 1; PACS2, phosphofurin acidic cluster sorting protein 2; PAH, pulmonary arterial hypertension; PDGF, platelet-derived growth factor; PDH, pyruvate dehydrogenase; PTPIP51, protein tyrosine phosphatase interacting protein 51; RyR2, ryanodine receptor 2; SERCA2b, sarco(endo)plasmic reticulum Ca2 + -ATPases 2b; Sig-1R, sigma-1 receptor; TMX-1, thioredoxin 1; UCP2, uncoupling protein 2; VAPB, vesicle-associated membrane protein-associated protein-B.

Mitochondrial dynamics, a major modulator of cellular metabolism, also plays a vital role in I/R through modulating mPTP opening [7, 48]. As expected, fragmentation of the mitochondrial network under ischemic conditions is related to increased cell death. However, the primary function of mitochondrial fission is to generate more mitochondria to meet energy demand required by cardiomyocytes in I/R [105]. Suppression of mitochondrial fission via inactivating Drp1 decreased HL-1 cell sensitivity to mPTP opening and reduced I/R-induced cell death. Additionally, mdivi-1 inhibited mitochondrial fission, promoting mitochondrial elongation and reduced cell death in cardiomyocytes under I/R. Mdivi-1 treatment also reduced infarct area in the heart from myocardial infarction (MI) mice [106]. Given that the inhibition of mitochondrial Ca^2+^ overload and respiratory chain activity in reperfusion relieves oxidative stress, Mfn1/2 knockout might be effective in alleviating cardiac dysfunction in short term. However, long term Mfn2 deletion was shown to result in inhibition of autophagy and mitochondrial fusion, en route to cardiac dysfunction [107]. Fig. 3 provides an overview of MAMs components involved in cardiac I/R injury while Table 1 summarizes their functions.

### Heart failure

Heart failure is a complicated and progressive disorder mainly caused by initial cardiac insufficiency and relies on a vicious cycle between neurohormonal activity and progressive cardiac remodeling [108–110]. Main MAM-resident elements served as potential therapeutic targets for heart failure are summarized in Fig. 3, and their specific roles are outlined in Table 1.

Overloading of mitochondrial Ca^2+^ by SR Ca^2+^ leakage via RyR2 channel in MAMs was found in post-myocardial infarcted mice models, contributing to altered mitochondrial activity and a deleterious elevation in mitochondrial Ca^2+^ concentration [111]. There might be a positive feedback loop between SR Ca^2+^ output and mitochondrial ROS, causing RyR2 leakage, intracellular Ca^2+^ raise and eventually apoptosis. Based on this view, blocking calcium-sensing receptor (CaSR) diminishes intercellular Ca^2+^ transmission, mitochondrial Ca^2+^ overload and apoptosis. Mitochondria Ca^2+^ influx was also shown to be interfered by FUNDC1, an OMM protein governing mitophagy. Patients diagnosed with heart failure present decreased FUNDC1 expression accompanied with poor SR-mitochondria contacts [112, 113]. Heart-specific FUNDC1 ablation mice exhibited mild interstitial fibrosis, compromised cardiac function and elevated apoptosis at baseline condition [114]. In addition, our group recently indicated that FUNDC1 may degrade IP_3_R3 though interacting with FBXL2 (receptor subunit of human SCF (SKP1/cullin/F-box protein) ubiquitin ligase complex) to sustain mitochondrial function and Ca^2+^ homeostasis. We further noted that FUNDC1 deficiency exacerbated cardiac remodeling and reduced systolic function in mice fed high-fat diet [67]. Another essential MAMs molecule involved in heart failure is Sig-1R, inhibition of which stimulates autophagy in cardiomyocytes under oxidative stress [115]. Hypertrophy and cardiomyocyte injury caused by angiotensin II were repressed by facilitated mitochondrial Ca^2+^ transfer and ATP production under Sig-1R stimuli [116]. Cardiomyocytes treated with highly specific Sig-1R agonists exhibited changes in Ca^2+^ transport, contractility and rhythmicity. Furthermore, with a high Sig-1R affinity, fluvoxamine ameliorated heart failure and cardiac dysfunction in rodent model of transverse aortic constriction [117, 118]. Furthermore, given the role of Ca^2+^ dysfunction in heart failure to trigger of mPTP opening, transgenic mice were established with induced, cardiac-specific overexpressed β2a subunit of L-type Ca^2+^ channel. These mice developed Ca^2+^ overload, spontaneous myocardial necroptosis, and heart failure. Intriguingly, deletion of cyclophilin D, a key regulator of mPTP and necroptosis, rescued this phenotype, suggesting necroptosis as a crucial component in the pathogenesis of heart failure [119, 120].

Maladaptive cardiac hypertrophy leads to heart failure and various functional deterioration, including aberrant mitochondrial dynamics. Substantial evidence has defined downregulated Mfn2 level in both in vivo and in vitro models of heart failure, for example, spontaneously hypertensive rats and hypertrophy induced by transverse aortic constriction [121]. Multiple studies have illustrated that upregulation of Mfn2 not only reversed ROS production and mitochondrial depolarization, but also retarded cardiomyocyte hypertrophy and pro-hypertrophic phenotype [39]. It is of interest to note that, norepinephrine evoked mitochondrial fission and cardiomyocyte hypertrophy by modulating Drp1 function [122]. Overexpression of devitalized Drp1 (DRP1K38A) in cultured neonatal mouse cardiomyocytes prevented norepinephrine-caused hypertrophy and mitochondrial network damage [123]. However, Drp1-dependent mitophagy played a prophylactic role against pressure overload-induced heart failure, consistent with the detrimental outcome of the Drp-1 inhibitor mdivi-1 in late stage of heart failure [124].

### Pulmonary arterial hypertension

PAH is vascular remodeling induced by phenotypical changes in pulmonary artery smooth muscle cells (PASMCs). PASMCs present a quiescent physiological contractile phenotype, which is converted to a hyperproliferative phenotype to favor small pulmonary vessel occlusion in PAH [125]. A number of factors have been indicated in the phenotypical switch in PASMCs (Fig. 3). Among which, uncoupling protein 2 (UCP2) plays an important role in the maintenance of PASMCs phenotype and serves as a selective regulator of MCU-dependent Ca^2+^ transfer from ER to mitochondria. Deficiency of UCP2 decreased mitochondrial Ca^2+^ level and Ca^2+^-dependent enzymatic activity in PASMCs, evoking mitochondrial damage including decreased mitochondrial biogenesis and mitophagy in endothelium [126, 127]. In addition, UCP2 knockout was shown to increase mitochondrial ROS production and diminish NO generation in endothelial cells, en route to vascular dysfunction. UCP2 ablation spontaneously produced PAH in mice, emphasizing the role of UCP2 in PAH disease development [128].

Moreover, phenotype alterations in PASMCs are linked with metabolic patterns transition, with altered ATP generation from mitochondrial oxidation to mainly rely on cytoplasmic glycolysis as the energy source [129]. Inhibition of mitochondrial oxidation reduced mitochondrial membrane hyperpolarization and ROS generation, elevating the threshold of mPTP opening and onset of apoptosis in PASMCs [130]. Pyruvate dehydrogenase (PDH) complex acts as a gatekeeper for glucose oxidation in the mitochondria, and this complex is a crucial factor of metabolic conversion in PASMCs, the function of which is suppressed by phosphorylation [130]. Ca^2+^ transmitted from the ER to the mitochondria modulates PDH phosphorylation through activation of PDH phosphatase and inhibition of PDH kinase, thereby boosting PDH activity and promoting glucose oxidation [131, 132]. Moreover, activation of PDH with dichloroacetic acid (a PDH kinase inhibitor) nullified hypoxia-induced phenotypic and metabolic switch in PASMCs.

Mitochondrial dynamics are also associated with phenotypic transition in PASMCs. Notably, PASMCs isolated from PAH patients showed mitochondrial fragmentation associated with elevated fission protein Drp1 and reduced fusion protein Mfn2 in MAMs (Table 1) [133]. Epigenetic upregulation of Drp1 adapter proteins, mitochondrial dynamics protein of 49 and 51 kDa (MiD49 and MiD51), fostered mitotic fission, impelling apoptosis resistance as well as pathological proliferation in PAH. Modulation of mitochondrial kinetics with Drp1 inhibitor mdivi-1 or Mfn2 overexpression abrogated the phenotypic switch in PASMCs [134]. Induction of cell cycle arrest in PASMACs from PAH patients restored pulmonary artery remodeling, alleviated pulmonary vascular resistance and right ventricular hypertrophy, as well as improved cell motility in rodent models [135].

### Vascular diseases

Except for respiratory system, phenotypic alteration in vascular smooth muscle cells (VSMCs) also plays a role in atherosclerosis and hypertension [136]. The roles of MAMs in vascular disease progression are summarized in Table 1. Notably, Moulis and colleagues noted the increased SR-mitochondria communication in VSMCs upon atherosclerotic lipid stimulation. Destruction of MAM contacts by PACS2 silencing promoted VSMCs apoptosis, one of the initial steps in atherosclerosis and plaque rupture through suppression of mitophagosome formation and mitophagy [137]. Furthermore, Mfn2 at MAMs appears to involve phenotypic shift in VSMCs. For example, VSMCs in balloon-injured arteries or spontaneously hypertensive rats exhibited resistance to apoptosis, higher proliferation rate and lower Mfn2 levels (Fig. 3). In contrast, overexpression of Mfn2 suppressed cell proliferation and neointima formation as well as restenosis induced by balloon injury in rat carotid arteries [138, 139]. Mfn2 overexpression also inhibited VSMCs proliferation evoked by oxidized low-density lipoprotein (ox-LDL) during atherogenesis [133].

More data are available on the role of mitochondrial dynamics in pathogenesis of systemic vascular diseases. During VSMCs shift from physiological quiescent contractile phenotype to the hyperproliferative phenotype which is resistant to apoptosis, reduction in Mfn2 expression at MAMs might be associated with the disturbance of mitochondrial network in proliferative cells. Moreover, platelet-derived growth factor (PDGF), as a known inducer of phenotypic changes in VSMCs, was shown to stimulate mitochondrial fission, VSMCs proliferation and migration [140, 141]. Besides, enrichment of Drp1 was found in calcified human carotid arteries, while Drp1-deficient heterozygotic mice were resistant to vascular calcification using an atherosclerosis model (Fig. 3) [134]. In addition, DRP1K38A transgenic mice displayed low levels of mitochondrial fission in vivo in association with restrained development of vascular injury-induced intimal hyperplasia [141].

## Conclusion

Acting as the most direct interacting bridge between the ER and mitochondria, MAMs are critical for integrating activities carried out by these two organelles, especially transmission and coordination of energy metabolism and regulation of apoptotic signals under chronic stress. Dysregulation of MAMs function is correlated with pathogenesis of cardiovascular diseases. Available evidence points out for possible biomedical applications of targeting MAMs and associated proteins as therapeutic strategies for the management of cardiovascular diseases.

As an interface between energy metabolism, proteostasis and cell fate control, the modulatory mechanisms and molecules participating in the maintenance of MAMs integrity and function are vital to identification of new therapeutic targets for prevention or treatment of heart diseases. Novel and more effective treatments are exigently required for cardiovascular diseases. MAMs-associated proteins and regulatory molecules may influence the progression of cardiovascular diseases directly, which is of great importance to improve patient survival. Further studies should focus on the identification of novel MAMs proteins and modulators, as well as the therapeutic potentials of these proteins and modulators in cardiovascular diseases.

## Supplementary information

check list

## Data Availability

Original data used for this report (although not applicable) will be made available upon request to the corresponding author.
